# Effects of Colchicine in a Rat Model of Diet-Induced Hyperlipidemia

**DOI:** 10.3390/antiox11020230

**Published:** 2022-01-25

**Authors:** Denisa-Mădălina Zălar, Cristina Pop, Elena Buzdugan, Bela Kiss, Maria-Georgia Ştefan, Steliana Ghibu, Doiniţa Crişan, Alexandra Buruiană-Simic, Adriana Grozav, Ileana Monica Borda, Cristina Ionela Mogoșan

**Affiliations:** 1 Department of Pharmacology, Physiology and Pathophysiology, Faculty of Pharmacy, “Iuliu Hațieganu” University of Medicine and Pharmacy, 400012 Cluj-Napoca, Romania; denisazalae@yahoo.com (D.-M.Z.); steliana.ghibu@umfcluj.ro (S.G.); cmogosan@umfcluj.ro (C.I.M.); 2 Department of Cardiology, Vth Medical Clinic, Faculty of Medicine, “Iuliu Hațieganu” University of Medicine and Pharmacy, 400012 Cluj-Napoca, Romania; buzelena@yahoo.com; 3 Department of Toxicology, Faculty of Pharmacy, “Iuliu Hațieganu” University of Medicine and Pharmacy, 400012 Cluj-Napoca, Romania; kbela@umfcluj.ro (B.K.); m.georgia.stefan@gmail.com (M.-G.Ş.); 4 Department of Pathology, Faculty of Medicine, “Iuliu Hațieganu” University of Medicine and Pharmacy, 400012 Cluj-Napoca, Romania; dcrisan@umfcluj.ro (D.C.); buruiana.alexandra@yahoo.com (A.B.-S.); 5 Department of Organic Chemistry, Faculty of Pharmacy, “Iuliu Hațieganu” University of Medicine and Pharmacy, 400012 Cluj-Napoca, Romania; adriana.ignat@umfcluj.ro; 6 Department of Medical Specialties, Faculty of Medicine, University of Medicine and Pharmacy “Iuliu-Hațieganu”, 400012 Cluj-Napoca, Romania; monicampop@yahoo.fr

**Keywords:** inflammation, colchicine, hyperlipidemia, oxidative stress, hepatotoxicity

## Abstract

Inflammation and hyperlipidemia play an essential role in the pathophysiology of endothelial dysfunction as well as atherosclerotic plaque formation, progression and rupture. Colchicine has direct anti-inflammatory effects by inhibiting multiple inflammatory signaling pathways. The purpose of our study was to evaluate colchicine activity in an animal model of hyperlipidemia induced by diet. A total of 24 male rats (wild type, WT) were divided into three groups: group one fed with a basic diet (BD) (WT + BD, *n* = 8), group two fed with a high-fat diet (HFD) (WT + HFD, *n* = 8)), and group three which received HFD plus drug treatment (colchicine, 0.5 mg/kg, i.p., daily administration). Total cholesterol, LDL-, HDL-cholesterol and triglycerides were determined. In addition, plasma transaminases, inflammation of oxidative stress markers, were measured. Tissue samples were evaluated using hematoxylin-eosin and red oil stain. At the end of the study, rats presented increased serum lipid levels, high oxidative stress and pro-inflammatory markers. The aortic histopathological section revealed that HFD induced signs of endothelial dysfunction. Colchicine treatment significantly resolved and normalized these alterations. Moreover, colchicine did not influence NAFLD activity score but significantly increased ALT and AST levels, suggesting that colchicine amplified the hepatocellular injury produced by the diet. Colchicine reduces plasma lipid levels, oxidative stress and inflammation markers and leads to more favorable histopathologic vascular and cardiac results. However, the adverse effects of colchicine could represent an obstacle to its safe use.

## 1. Introduction

Cardiovascular diseases (CVD) are one of the major causes of death in the Western world, representing the most important mortality factor worldwide, leading to around 20 million deaths annually, accounting for 31.5% of all deaths [1]. Studies have shown that over 19% of adults in Romania have three or more vascular risk factors, including dyslipidemia, with a prevalence of dyslipidemia of over 46% [2,3]. Based on World Health Organization (WHO) global statistics, dyslipidemias are an important cause of ischemic heart disease and cerebrovascular disease, accounting for nearly 2.6 million deaths globally each year [1]. Dyslipidemia can result in structural and functional vascular modifications, leading to cardiac consequences such as coronary atherosclerosis and ischemic heart disease (IHD) [4,5,6].

Atherosclerosis was historically defined by the retention of lipids in the arterial wall [7,8,9]; however, newer research has changed this paradigm revealing that inflammation is a crucial factor in vascular pre-atherosclerotic modifications caused by dyslipidemia [10,11,12]. In recent studies, high CRP levels (C-reactive protein) are among the first indicators of vascular modifications and cardiovascular events. Locally, pro-inflammatory cytokines are released by macrophages [13,14]. Interleukine-1β (IL-1β), interleukine-6 (IL-6), tumor necrosis factor-α (TNF-α) and CRP could represent new therapeutic targets for anti-inflammatory treatment in endothelial dysfunction for reducing the risk of atherosclerotic cardiovascular disease (ASCVD) [15,16]. In addition, hyperlipidemia can increase the production of free radicals from the mitochondrial electron transport chain or nicotinamide adenine dinucleotide phosphate (NADPH) activation oxidase [17]. Malondialdehyde or 4-hydroxynonenal, final products of lipid peroxidation, produced either by the high levels of reactive oxygen species (ROS) or because of the failure of the antioxidant system, may induce protein damage by reactions with lysine, histidine or cysteine groups [18,19].

Colchicine, a microtubule polymerization inhibitor, acts by: (1) inhibiting neutrophils diapedesis and production of inflammatory cytokines in the endothelium [20]; (2) inhibiting the release of leukotriene B4 (LTB4), an important neutrophil activator [20]; (3) modulating TNF-α activity [21]; (4) inhibiting neutrophil secretion process. In addition, colchicine may stabilize membranes by decreasing lipid peroxidation [22].

Colchicine is an alkaloid of *Colchicum autumnale* L. with specific anti-inflammatory action in gout attacks. The probable mechanism is considered to reduce the formation of urate crystals and decrease phagocytosis and the consequent inflammatory reaction. It is thought that colchicine may reduce the serum level of IL-1β by inhibiting the NLRP (nod-like receptor pyrin 3) inflammasome pathway [23]. Chronic therapy with colchicine can be associated with liver enzymes elevations. Colchicine may inhibit microtubule or spindle formation and generate mitotic arrest, thus inducing hepatotoxicity because of increased hepatic metabolism [24].

Thus, we aimed at investigating the effects of colchicine on lipid profile, inflammation markers and vascular, cardiac and hepatic tissues in a model of diet-induced dyslipidemia in rats.

## 2. Materials and Methods

### 2.1. Animal Model

Sprague-Dawley WT rats (*n* = 24, male, 300 ± 20 g) were purchased from “Cantacuzino” National Medico-Military Institute for Research and Development, Bucharest, Romania.

NIH (National Institute of Health) guidelines were followed for all procedures. Animal Ethics Committee of the University of Medicine and Pharmacy “Iuliu Hatieganu” Cluj-Napoca approved the experiment (Permit number: M20150505).

Animals were kept in standard conditions (room temperature 23 ± 1 °C and 55 ± 5% relative humidity, 12 h light/dark cycle). Food and water were provided ad libitum.

Colchicine used in the experiment was purchased from Sigma-Aldrich, USA, batch number: SLBQ8131V.

The types of diet used were basic diet (BD) and high-fat diet (HFD) purchased and prepared as previously described [25].

Rats were included randomly into three groups: (1) WT + BD group (wild type rats fed with a standard diet, *n* = 8); (2) WT + HFD (wild type rats fed with HFD, *n* = 8); (3) WT + HFD + Col (wild type rats fed with HFD and treated with colchicine 0.5 mg/kg, intraperitoneally (i.p), daily administration, *n* = 8) [26].

The total duration of the study was nine weeks and during this time rats received either standard or HFD, while after four weeks of HFD, colchicine treatment was started, for 5 weeks.

After 9 weeks, blood, liver, aorta and myocardial tissue were sampled, as previously described [25].

### 2.2. Liver and Plasma Lipids Profile

Aminotransferases (aspartate aminotransferase (AST), alanine aminotransferase (ALT)) and plasma lipids profile (total cholesterol (TC), triglyceride (TG), low-density lipoprotein cholesterol (LDL-C), high-density lipoprotein cholesterol (HDL-C)) were determined, as previously described [25].

### 2.3. Inflammation Markers

The concentrations of hs-CRP, IL-1β, IL-6 and TNF-α in plasma were measured as previously described [25].

### 2.4. Oxidative Stress Markers

The analysis of reduced glutathione (GSH), oxidized glutathione (GSSG) as well as malondialdehyde (MDA) was performed on plasma samples obtained from whole blood (treated with EDTA), after centrifugation.

The first step in the analysis of GSH was the deproteinization of 150 µL of plasma with metaphosphoric acid (10%, m/v). The samples were then diluted with EDTANa_2_ in phosphate buffer (pH = 8, 0.1%), followed by incubation with ortho-phthalaldehyde (OPA). The obtained GSH derivative was quantified by liquid chromatography. For the analysis of GSSG, the samples were deproteinized, incubated with N-ethylmaleimide, diluted with 0.1 M NaOH, and derivatized with OPA. Similarly, the obtained GSSG derivative was assayed by liquid chromatography.

The chromatographic methods employed for GSH and GSSG analysis were based on ultra-performance liquid chromatography (Waters Acquity UPLC) coupled with fluorescence detection (Waters Acquity FLD, λ_exc_ = 350 nm, λ_em_ = 420 nm). An HSS T3 Acquity UPLC column (1.8µm, 2.1 × 100 mm) was used to separate the analytes and the mobile phase consisted of a mixture of 25 mM Na_2_HPO_4_ and MeOH.

The analysis of total MDA in plasma required: (1) an alkaline hydrolysis step, with 6 M NaOH at 60 °C; (2) perchloric acid (35%) deproteinization; (3) derivatization of MDA with 2,4-dinitrophenylhydrazine; (4) extraction of the obtained derivative in hexane; (5) evaporation of the organic phase to dryness; (6) dissolution of the residue in mobile phase; (7) injection of the obtained solution in the UPLC-PDA system (λ = 307 nm). Diacetone alcohol was used as internal standard and the separation of analytes was achieved using a BEH C18 column (50 mm × 2.1 mm i.d., 1.7 mm), and gradient elution with a mixture of formic acid (1%) and acetonitrile.

Data acquisition and processing were performed using Empower 2 software (Waters, USA) for all chromatographic assays.

### 2.5. Histopathological Analysis of Liver, Heart and Aorta

Paraffin sections of the aortic arch, liver and heart (4–5 µm) were prepared as previously described [25].

### 2.6. Echocardiography

At the end of the treatment period (9 weeks) rats were subjected to anesthesia (30 mg/kg ketamine and 0.5 mg/kg xylazine, i.m.) and transthoracic echocardiography was performed in the left lateral position by using a commercially available echocardiograph, including a 7.5 MHz electric transducer (Ultrasonix, Boston, MA, USA), as previously shown [27].

Structural and functional echocardiographic parameters were acquired using the leading-edge method of the American Society of Echocardiography [28]. The main parameters that were evaluated include: interventricular septum thickness (IVS), posterior wall thickness (PW), end-diastolic diameter (EDD), end-systolic diameter (ESD), ejection fraction (EF) determined by the Simpson rule, left ventricular mass (LV mass), cardiac output (CO) and heart rate.

### 2.7. Statistical Analysis

For the statistical analysis, SPSS 26.0 (Statistical Package for the Social Science, SPSS Inc., Chicago, IL, USA) software was used and figures were created using GraphPad Prism 5. Experimental data were presented as mean ± SD. Mann–Whitney tests were used for ordinal/semiquantitative variables, Fisher tests for qualitative variables and one-way ANOVA was used for multi-group comparisons. For quantitative variables, data comparison used Student’s t-tests. A value of *p* < 0.05 was considered statistically significant.

## 3. Results

### 3.1. Effects of Colchicine on Plasma Lipid Profile

**Figure 1:**
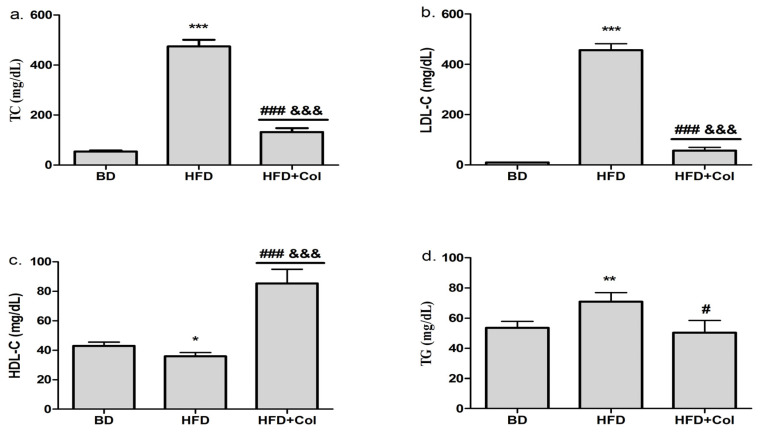
High-fat diet and colchicine effects on lipid profile: BD—basic diet; HFD—high-fat diet; HFD + Col—high-fat diet and colchicine; (**a**) total cholesterol (TC), (**b**) low-density lipoprotein cholesterol (LDL-C), (**c**) high-density lipoprotein cholesterol (HDL-C), (**d**) triglycerides (TG); * *p* < 0.05; ** *p* < 0.01; *** *p* < 0.001 HFD vs. BD group; ^#^ *p* < 0.05; ^###^ *p* < 0.001 HFD + Col vs. HFD group; ^&&&^ *p* < 0.001 HFD + Col vs. BD group.

At the end of the HFD period, we observed that TC, LDL-C (*p* < 0.001) and TG (*p* < 0.01) were considerably increased in the HFD group compared to the BD group (Figure 1a,b,d), while HDL-C levels decreased in HFD group (Figure 1c). Colchicine administration showed beneficial effects on lipid profile, significantly decreasing TC (*p* < 0.001), LDL-C (*p* < 0.001), and TG (*p* < 0.05) levels, while markedly increasing HDL-C (*p* < 0.001) (Figure 1c).

### 3.2. Anti-Inflammatory Effects of Colchicine

**Figure 2:**
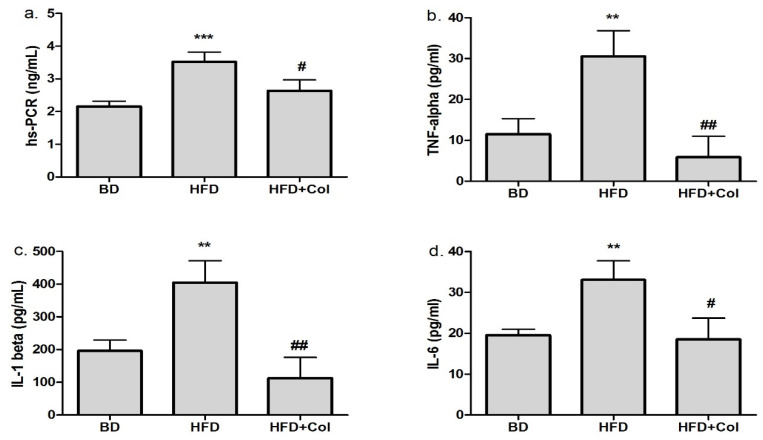
Colchicine effects on inflammation biomarkers levels: BD—basic diet; HFD—high-fat diet; HFD + Col—high-fat diet and colchicine; (**a**) high sensitivity reactive protein C (hs-PCR), (**b**) tumor necrosis factor-alpha (TNF-alpha), (**c**) interleukine-1 beta (IL-1 beta), (**d**) interleukine-6 (IL-6); ** *p* < 0.01; *** *p* < 0.001 HFD vs. BD group; ^#^ *p* < 0.05; ^##^ *p* < 0.01 HFD + Col vs. HFD group.

In the group fed with a high-fat diet, we observed a marked increase regarding the inflammation biomarkers (hs-PCR, TNF-α, IL-1β and IL-6), while colchicine treatment significantly decreased the above-mentioned biomarkers, compared to the HFD group. No significant difference was observed between BD and HFD + Col groups concerning inflammation markers (Figure 2).

### 3.3. Antioxidant Effects of Colchicine

**Figure 3:**
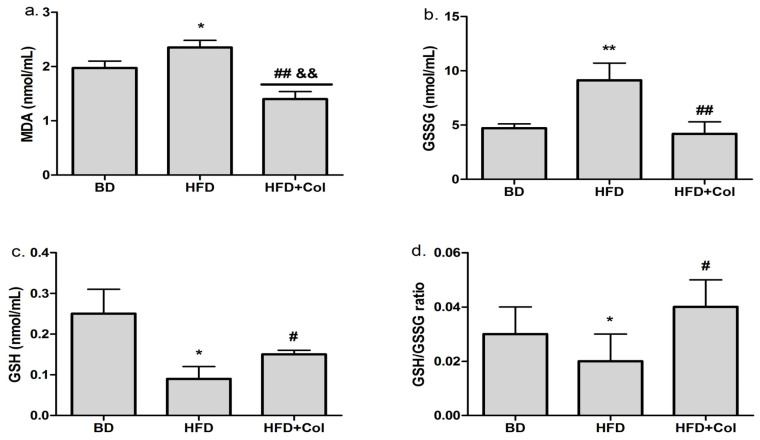
Colchicine effects on oxidative stress biomarkers: BD—basic diet; HFD—high-fat diet; HFD + Col—high-fat diet and colchicine; (**a**) malondialdehyde (MDA), (**b**) reduced glutathione (GSH), (**c**) oxidized gluthatione (GSSG), (**d**) GSH/GSSG ratio; * *p* < 0.05; ** *p* < 0.01 HFD vs. BD; ^#^ *p* < 0.05; ^##^ *p* < 0.01 HFD + Col vs. HFD; ^&&^ *p* < 0.01 HFD + Col vs. BD group.

As shown in Figure 3, the oxidative stress markers (MDA, GSSG) were higher (*p* < 0.05) in the group fed with HFD in comparison with the BD group (Figure 3a,b). GSH and GSH/GSSG ratios were significantly lower (*p* < 0.05) in the HFD group in comparison with the BD group (Figure 3c,d).

Colchicine treatment led to a considerably decreased level of oxidative stress biomarkers (MDA and GSSG) (*p* < 0.01) when compared to the HFD group (Figure 3a,b); at the same time, GSH and GSH/GSSG ratio significantly increased (*p* < 0.05) (Figure 3c,d). Moreover, colchicine treatment significantly decreased MDA levels, as they were even lower compared to the BD group (Figure 3a).

### 3.4. Colchicine Effects on Liver Enzymes

**Figure 4:**
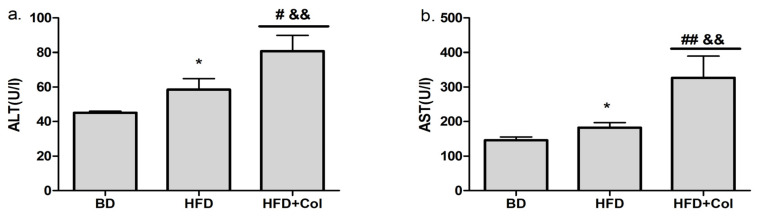
Colchicine effects on liver enzymes: BD—basic diet; HFD—high-fat diet; HFD + Col—high-fat diet and colchicine; (**a**) alanine aminotransferase (ALT), (**b**) aspartate aminotransferase; * *p* < 0.05 HFD vs. BD; ^#^ *p* < 0.05; ^##^ *p* < 0.01 HFD + Col vs. HFD; ^&&^ *p* < 0.01 HFD + Col vs. BD group.

Regarding liver function, we observed a higher level of liver enzymes (ALT and AST) (*p* < 0.05) in groups fed a high-fat diet, compared to groups that received a basic diet (Figure 4a,b). The treatment with colchicine led to significantly increased transaminases levels compared to both HFD and BD groups (Figure 4a,b).

### 3.5. Histopathological Changes in Liver, Heart and Artery Tissue

Following histopathological analysis, we observed that the high-fat diet led to a considerable infiltration of fat in the hepatic cells compared to the groups that were fed with the basic diet.

**Figure 5:**
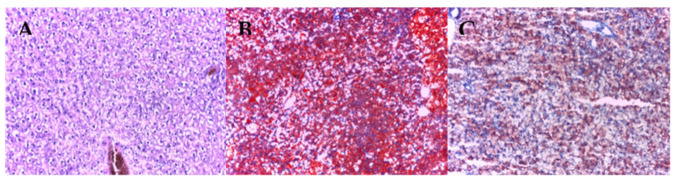
Colchicine effects on liver sections stained with Red Oil (200×). The fat infiltration in the hepatic cells is indicated by the red coloration: (**A**) WT + BD; (**B**) WT + HFD; (**C**) WT + HFD + Col; BD—basic diet; HFD—high-fat diet; HFD + Col—high-fat diet and colchicine.

**Figure 6:**
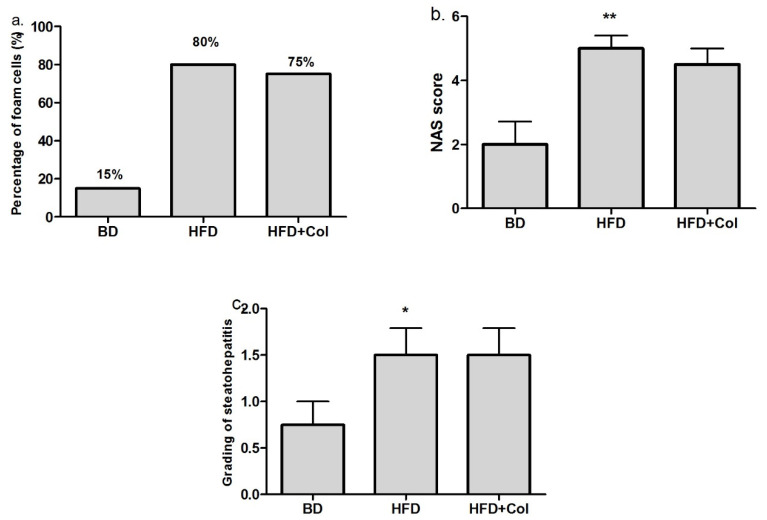
Colchicine effects on histological features, (**a**) Percentage of fat infiltration in the liver, (**b**) non-alcoholic fatty liver disease activity score (NAS), (**c**) grading of steatohepatitis. 1—mild, 2—moderate, 3—severe; BD—basic diet; HFD—high-fat diet; HFD + Col—high-fat diet and colchicine; ^*^ *p* < 0.05; ** *p* < 0.01 HFD vs. BD.

Colchicine administration slightly reduced the fat infiltration in liver cells compared to the HFD group (Figure 5 and Figure 6a).

The NAFLD (non-alcoholic fatty liver disease) activity score (NAS) was established based on histological features (steatosis, lobular inflammation, hepatocyte ballooning). The NAS score markedly increased (*p* < 0.01) in the HFD group compared to the BD group, while colchicine administration did not produce statistically significant changes in the NAS score (Figure 6b). The degree of steatohepatitis was mild in BD groups, and moderate in the HFD group (*p* < 0.05), whereas administration with colchicine did not produce changes regarding the steatohepatitis grading when compared to the HFD group (Figure 6c).

**Figure 7:**
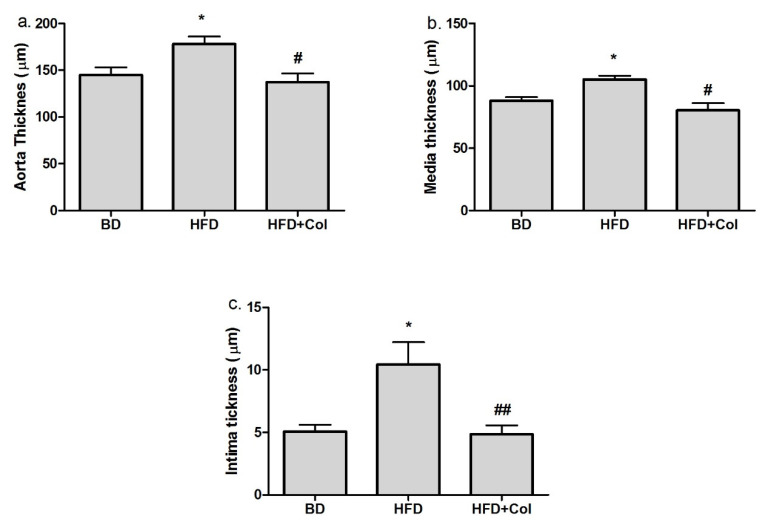
Colchicine effects on aortic histopathological sections: (**a**) aorta tickness, (**b**) media tickness, (**c**) intima thickness. BD—basic diet; HFD—high-fat diet; HFD + Col—high-fat diet and colchicine; * *p* < 0.05 HFD vs. BD; ^#^ *p* < 0.05; ^##^ *p* < 0.01 HFD + Col vs. BD.

Regarding the aortic histopathological section, we observed that the high-fat diet induced characteristic changes revealing aorta, media and intima thickening (*p* < 0.05) (Figure 7), fat infiltration in liver and aortic sections stained with H&E and oil red, a disorganized structure of the nucleus, smooth muscle cells and elastic fibers migration.

The treatment with colchicine decreased the aorta (*p* < 0.05), media (*p* < 0.05) and intima (*p* < 0.01) thickness (Figure 7).

### 3.6. Colchicine Effects on Echocardiographic Parameters

Effects of both high-fat diet and colchicine treatment are shown in Table 1. HFD modified both structural and functional cardiac parameters. Concerning structural parameters, HFD significantly increased end-diastolic diameter (EDD) and end-systolic diameter (ESD), and regarding functional parameters, HFD led to a significant decrease in ejection fraction (EF). Treatment with colchicine significantly improved both structural (EDD and ESD) and functional (EF) parameters. The HFD group presents a higher LV mass compared to the BD group. In the treatment group (HFD + Col), LV mass is slightly lower compared to the HFD group, but the difference is not statistically significant. There is a slightly higher HR in the BD group compared to the other groups, however there is no statistical significance concerning HR between groups. CO is significantly lower in the HFD group compared to the BD group. Colchicine treatment significantly improves CO compared to HFD. There is no statistical difference between CO in HFD + Col and BD groups.

## 4. Discussion

Dyslipidemia represents one of the major causes of premature coronary atherosclerosis, which leads to ischemic heart disease (IHD). Hyperlipidemia, along with high LDL-C and low HDL-C serum levels, are key factors in the incidence of coronary heart disease, morbidity, and cerebrovascular mortality [29,30].

Our study objective was to determine the potential favorable effects of colchicine on plasma lipid profile and the anti-inflammatory and antioxidant effects of colchicine.

In this research, we successfully induced hyperlipidemia by providing Sprague-Dawley (wild type, WT) rats with a high-fat diet for nine weeks, as previously described [25]. As reported in the literature, there is an interrelationship regarding the high-fat diet, hypercholesterolemia, and the inflammatory process [25,29,31,32,33].

HFD induces hyperlipidemia proven by increased plasma concentration of CT, LDL-C, and TG, which initiates an inflammatory response, supported through increased inflammatory biomarkers (hs-PCR, TNF-α, IL-1β, IL-6) compared to the BD group.

Colchicine is an alkaloid of *Colchicum autumnale* L. with specific anti-inflammatory action in gout attacks. The probable mechanism is considered to reduce the formation of urate crystals and decrease the phagocytosis and the consequent inflammatory reaction, acting mainly on neutrophils, monocytes and macrophages, reducing their chemotaxis and releasing different pro-inflammatory cytokines [34]. It is thought that colchicine may decrease the serum level of IL-1β and IL-18 (essential pro-inflammatory cytokines downstream IL-6) by inhibiting the NLRP Inflammasome pathway [23,35,36]. It may be due to the interruption of microtubule-dependent transport of mitochondria to the endoplasmic reticulum. In an animal model of pulmonary arterial hypertension, colchicine administration suppressed smooth muscle cell proliferation, elevated apoptosis and diminished the protein expression of inflammation (TNF-α and NF-κB) [34]. LoDoCo (Low-Dose Colchicine for Secondary Prevention of Cardiovascular Disease) was the first prospective study of 523 patients with stable coronary heart disease to analyze the effect of colchicine on long-term cardiovascular prognosis. At a median follow-up of 3 years, the addition of 0.5 mg to standard preventive therapy decreased the risk of adverse cardiovascular events. The hypothesis of this study was based on the anti-inflammatory action of colchicine, by suppressing pro-inflammatory cytokines and preventing cholesterol crystal-induced neutrophil-mediated inflammation implicated in atherosclerosis [37]. In addition, colchicine significantly decreased hs-CRP levels, stimulated by the release of IL-6 from activated monocytes. High-sensitivity CRP is an acute phase reactant (specific inflammation marker) that can be used as a prognostic factor for coronary heart disease, risk assessment, and the evaluation of therapy response in CVD [38].

In this paper, we observed that colchicine administration decreases plasma levels of hs-CRP and inflammatory mediators (IL-1β, IL-6, TNF-α), and also considerably reduces the plasma levels of TC, LDL-C and TG while increasing the HDL-C level. Our outcomes are sustained by the results of Aernoud T et al., showing that short-term administration of colchicine may lead to a decrease of the inflammatory markers hs-CRP and IL-6 in patients with chronic CAD [39].

The high-fat diet led to impaired liver function proved by elevated ALT and AST levels, highlighting the presence of hepatocellular lesions [40]. Colchicine treatment induced hepatotoxicity, supported by considerably high levels of hepatic enzymes (AST and ALT) in rat liver, indicating that colchicine exacerbated the hepatotoxicity induced by the HFD [24].

MDA is a standard biomarker in the assessment of oxidative stress, its plasma levels are an indicator regarding the degree of lipid peroxidation. Increased levels of MDA have been reported in animal models of cardiovascular disease [27,39,40,41]. The interplay between ROS, membranes, and lipids, increases membrane permeability, initiating cell death processes, such as apoptosis. GSH is involved in many metabolic processes, presenting various roles at the cellular level such as eliminating free radicals directly and indirectly through enzymatic reactions [27,41,42]. An imbalance between free radicals and the antioxidant system causes inadequate detoxification of the reactive oxygen species. The accumulation of free radicals increases oxidative stress as shown in hypercholesterolemia conditions [19,43].

Our results showed that the high-fat diet considerably reduced the GSH and GSH/GSSG ratio levels compared to the BD group, at the same time increasing MDA and GSSG plasma levels. These findings indicate that HFD reduces the antioxidant capacity, causing an increase in free radicals, inducing overproduction of MDA [40]. Our data are consistent with previous studies published in the literature and indicate that HFD promotes intracellular oxidative stress [41].

According to our data, colchicine administration increased GSH and GSH/GSSG ratio levels and decreased MDA and GSSG levels, indicating that colchicine may decrease oxidative stress and enhance antioxidant capacity. As previously described in the literature, colchicine administration induces a significant reduction in oxidant molecules and an increase in antioxidant capacity [20,22].

In our study, the histopathological examination of aortic tissue showed that the rats fed with the high-fat diet exhibited structural and functional changes in tissues, such as aortic intima-media thickening, fat infiltration in liver and aortic sections, a disorganized structure of the nucleus, smooth muscle cells and elastic fibers migration, and all these features are thought to be characteristic changes leading to atherosclerosis onset [44]. Furthermore, the histopathological analysis also revealed a considerable infiltration of lipid droplets in the hepatic cells and the progress of steatohepatitis grading from mild to moderate, and all these factors are directly involved in the onset of atherosclerosis [44]. The aortic histopathological section showed that the treatment with colchicine decreased the intima-media thickening, improving the aortic wall and slightly decreasing the hepatic lipid accumulation without influencing the NAS score. These results suggest that colchicine could influence the initiation and evolution of atherosclerosis (AS), reducing the risk of AS to a large extent.

Beneficial cardiovascular effects of colchicine are also supported by our echocardiography results. In our experiment, HFD increased both structural (EDD, EDS, LV mass) and functional (EF, CO) echocardiographic parameters. Colchicine treatment improves EDD and ESD and corrects ventricular performance, thus resulting in an improved cardiac output. Similarly, Fujisue et al. [45] observed that colchicine improved EDD and EF and overall survival rate in a mouse model of acute myocardial infarction. Studies on human patients diagnosed with acute and chronic coronary syndromes revealed that the daily use of colchicine was both safe and effective at reducing the risk of cardiovascular death and myocardial infarction [46]. A recent clinical trial (COLCOT—colchicine cardiovascular outcomes trial) revealed that colchicine significantly improved cardiovascular function after myocardial infarction [47]. Tubulin disruption and inhibition of microtubule polymerization, the main mechanism of action of colchicine, leads to beneficial cardiovascular effects successfully used in pericarditis, atrial fibrillation and myocardial infarction, but recent studies have highlighted the beneficial effects of colchicine on dyslipidemia-induced inflammation. Thus, an important use of colchicine is the prophylactic effects concerning atherosclerotic plaque formation and rupture, to prevent cardiovascular events such as myocardial infarction and the consequences that result from it [48].

In conclusion, our results showed that colchicine reduces the inflammatory response and enhances the antioxidant capacity in groups fed with a high-fat diet. Colchicine could represent a potential therapeutic target for the treatment of hyperlipidemia, due to its possible beneficial effects, favorably influencing a series of biomarkers that generate and maintain the atherosclerotic process diminishing the pro-inflammatory response and oxidative stress. The advantages of colchicine compared to other drugs include: the well-defined safety profile, the high availability, as it is already used in therapy, the acceptability of the route of administration, as it is administered orally, and the low price. Nevertheless, the benefit versus risk ratio and the adverse events should be carefully evaluated.

Further studies are needed to establish whether or not colchicine treatment results in a lower cardiovascular risk.

## Figures and Tables

**Table 1:** Effects of a high-fat diet and colchicine treatment on echocardiography parameters.

Echocardiography Parameter	BD (*n* = 8)	HFD (*n* = 8)	HFD + Col (*n* = 8)
IVS (interventricular septum) (mm)	1.43 ± 0.34	1.43 ± 0.25	1.46 ± 0.18
PW (posterior wall) (mm)	1.70 ± 0.30	1.70 ± 0.26	1.75 ± 0.24
EDD (end-diastolic diameter) (mm)	6.63 ± 0.95	7.41 ± 0.69 *	7.10 ± 0.42 ^#^
ESD (end-systolic diameter) (mm)	4.17 ± 0.79	5.25 ± 0.71 *	4.73 ± 0.47 ^#^
LV mass (g)	1.14 ± 0.06	1.24 ± 0.01 *	1.21 ± 0.08
EF (ejection fraction) Simpson rule (%)	72.87 ± 7.81	66.61 ± 5.70 *	74.59 ± 4.16 ^#^
CO (cardiac output) (mL)	124.16 ± 5.12	113.19 ± 2.41 *	129.22 ± 5.61 ^#^
Heart rate (bpm)	258.80 ± 45.64	189.63 ± 28.37	233.17 ± 23.15

BD—basic diet; HFD—high-fat diet; HFD + Col—high-fat diet and colchicine; * *p* < 0.05 HFD vs. BD; ^#^ *p* < 0.05 HFD + Col vs. HFD.

## Data Availability

The data presented in this study are available in this manuscript.
